# Time trends in socio-economic inequalities in the lack of access to dental services among children in Spain 1987-2011

**DOI:** 10.1186/s12939-015-0132-8

**Published:** 2015-01-31

**Authors:** Jaime Pinilla, Miguel A Negrín-Hernández, Ignacio Abásolo

**Affiliations:** Departamento de Métodos Cuantitativos en Economía y Gestión, Universidad de Las Palmas de Gran Canaria, Las Palmas, Spain; Departamento de Economía Aplicada y Métodos Cuantitativos, Facultad de Economía, Empresa y Turismo and IUDR, Universidad de La Laguna, Campus de Guajara, 38071 La Laguna, Tenerife, Spain

**Keywords:** Inequalities in lack of access, Dental services, Children, Spanish National Health System

## Abstract

**Introduction:**

Adult oral health is predicted by oral health in childhood. Prevention improves oral health in childhood and, consequently in adulthood, so substantial cost savings can be derived from prevention. The burden of oral disease is particularly high for disadvantaged and poor population groups in both developing and developed countries. Therefore, an appropriate and egalitarian access to dental care becomes a desirable objective if children’s dental health is to be promoted irrespective of socioeconomic status. The aim of this research is to analyse inequalities in the lack of access to dental care services for children in the Spanish National Health System by socio-economic group over the period 1987–2011.

**Methods:**

Pooled data from eight editions of the Spanish National Health Survey for the years 1987–2011, as well as contextual data on state dental programmes are used. Logistic regressions are used to examine the related factors to the probability of not having ever visited the dentist among children between 6 and 14 years old. Our lack of access variable pays particular attention to the socioeconomic level of children’s household.

**Results:**

The mean probability of having never been to the dentist falls considerably from 49.5% in 1987 to 8.4% in 2011. Analysis by socioeconomic level indicates that, in 1987, the probability of not having ever gone to the dentist is more than two times higher for children in the unskilled manual social class than for those in the upper non-manual social class (odds ratio 2.35). And this difference is not reduced significantly throughout the period analysed, rather it increases as in 1993 (odds of 2.39) and 2006 (odds of 3.03) to end in 2011 slightly below than in 1987 (odds ratio 1.80).

**Conclusion:**

There has been a reduction in children’s lack of access to dentists in Spain over the period 1987–2011. However, this reduction has not corrected the socioeconomic inequalities in children’s access to dentists in Spain.

## Introduction

There are several reasons to analyse socioeconomic inequalities in the lack of access to children’s dental care services. First, oral health status may have an impact on health-related quality of life [1]. In fact, lack of appropriate oral health can lead not only to aesthetic problems related to appearance –like those generated by obesity or baldness- [2], but also to functional problems in adulthood like chewing, eating and having social relationships. It can even contribute to the development of some severe illnesses like coronary heart diseases or atherosclerosis (see for example Joshipura et al. [3], Scannapieco et al. [4] or Meurman et al. [5]). Second, oral diseases have a high prevalence and incidence in all regions of the world, with the greatest burden of oral diseases being on disadvantaged and socially marginalized populations [6]). Third, adult oral health is predicted by oral health in childhood, as well as by childhood socioeconomic advantages or disadvantages [7]. There is evidence of a relationship between socioeconomic conditions and early childhood caries, as this condition is more frequently found in children that live in poor economic conditions (see for example Davies [8], Rajab and Hamdan [9]). In addition, one of the most common chronic diseases in childhood is early childhood caries [10]. Fourth, prevention improves oral health in childhood and substantial cost savings can be derived from this [11]; prevention involves a combination of community, individual and professional measures. Regarding the latter, it consists of visiting the dentist at least once a year despite having no symptoms at all, in order to prevent dental pathologies and to educate children about how to take care their teeth. Fifth, treatment of oral disease is very costly, to the extent that it is the fourth most expensive disease to treat in most industrialized countries [12].

Therefore, the importance of socioeconomic level as a potential driver of both oral health and access to dental care has led us to analyse the inequalities in the lack of access to dental care services by socioeconomic groups. Our research is undertaken in the Spanish context and for a sufficiently long period (1987–2011), on the basis that, rather than identifying the extent of inequality in the lack of access at a particular point in time, it is more informative and relevant to analyse whether such a situation remains or whether there is a trend in the access pattern over time.

Spain has a National Health Care System characterised by universal coverage and tax funding; responsibility for healthcare provision lies with each of the seventeen *Comunidades Autónomas* (hereafter, regions). These regions are a consequence of the progressive political decentralisation process undertaken since the seventies.

A remarkable objective of the Spanish National Health Service has been to achieve a wider coverage in dental care services for children. However, the time and way in which this aim has been undertaken has varied among regions. In the early 90s, Pais Vasco and Navarra were the first two regions that developed the so called Children’s Dental Care Programme PADI (Programa de Atención Dental Infantil). Such programmes widened the provision of dental care with new services and improved children’s access possibilities through agreements with private dental clinics. Patients could choose between the corresponding public centre or the nearest private dentist enrolled in the programme (the latter being publicly paid on a per capita basis). The final aim of the PADIs programmes was to promote the utilisation of dental care services. During the following years, the PADIs programmes were progressively generalised across other regions: Andalucía in 2002, Murcia in 2003, Aragón, Baleares y Extremadura in 2005, and Canarias in 2008. Castilla-Leon and Castilla-La Mancha in 2003 and 2004 respectively and Madrid in 2010, chose a mixed model, public in the first instance but susceptible to being referred to the private sector for particular dental treatments. The remaining regions just maintained the conventional public model not having additional dental care services for children [13].

Therefore, the progressive implementation of different dental care programmes across Spain had as their main objective to achieve greater access to dental care services for children irrespective of the socioeconomic level of their households. In line with this, Cortés and Llodra [14] indicate, first, the need to guarantee free of charge access to preventive and treatment dental services for children between 6 and 15 years old as a strategy to achieve good levels of oral health in maturity. Second, the report suggests introducing reforms that would reduce inequalities in access to dental services irrespective of characteristics like socioeconomic level or the geographical area of residence. A crucial question is, therefore, whether in the past twenty five years there has been an improvement in access to dental care services and whether it is independent of the socioeconomic characteristics of children’s households.

Some studies have tackled the analysis of inequality in access to dental care services in Spain considering the use of dental care services across socioeconomic groups, normally in a cross-sectional setting. Stoyanova [15] finds that both income-inequality and income-inequity exist in dental care using an adult sample from 1997. Tapias-Ledesma et al. [16] in a study of children aged 3–15 for 2001 conclude that children in households with a lower family income and parents with the lowest educational level register a significantly lower use of dental services the year previous to the survey. Other studies have also considered the effect of contextual variables such as the type of dental care model offered in different regions in use of dental care services. For instance, García-Gómez [17] shows evidence that indicates that the infant oral care programme promoted in the País Vasco region is associated with an increase in the probability of children visiting the dentist. Pinilla and González [18] analyse the impact on equity in the long run of different infant oral care programmes. They estimate the probability of visiting the dentist together with the number of visits, aiming to compare regions with the programme and regions without the programme. They conclude that in comparison with regions without infant oral care programmes, those regions that do have it, the probability of using it is greater and independent of household income. Also, Barriuso and Sanz [19] analyse the individual and contextual variables associated with the use of oral health services by a population aged 6 to 15 and adjusted by need, which is approached by different measures of dental disease. They conclude that the use of oral health services is lower than recommended and is positively correlated with socioeconomic level and with living in regions having an infant oral care programme of 10 or more years standing.

To our knowledge, there have not been any studies that analyse this topic for a sufficiently long period to ascertain whether any inequalities in access persist. Therefore, the aim of this research is to analyse inequalities in the lack of access to dental care for children in Spain by socio-economic groups over the period 1987–2011 (and examine the major determinants of lack of access to child dental care).

## Methods

### Data source/s

Data at the individual level comes from eight editions of the Spanish National Health Survey (SNHS) conducted in Spain in 1987, 1993, 1995, 1997, 2001, 2003, 2006 and 2011. These repeated cross-sectional surveys were specifically established to collect data on adult and child health indicators that are representative at the national state level. The SNHS contemplates a sample of non-institutionalized children aged from 0 to 15 years old with the exception of 2011 when the sample was from 0 to 14, distributed throughout the 17 regions of Spain (the non-institutionalized population was approximately 99.76% in 1989 and 99.85% in 2011 of total population [20]). Ceuta and Melilla (two small Autonomous Cities –not regions- with no power in the management of health care provision) were excluded from the analysis as information on children was not gathered from 1987–1997. Due to their relatively small sample size, data from the 1995 and 1997 surveys were analysed jointly. Details of the methodology, sample design, sample size and sampling procedure together with the anonymous microdata of the eight National Health Survey editions are publicly available [21]. Regional rate of dentists per 100.000 inhabitants data come from the Spanish National Statistics Institute [22].

After a generational classification of individuals based on reported age and year of the survey, we have combined the micro-data of the eight published editions of the SNHS. The combined file contains common variables across all of the SNHSs (or harmonised variables like household social status), which provides us with a sufficiently homogenous series to undertake pooled analysis. An average effective sample of 89% households has been obtained from the initials dwellings selected for all years 1987–2011. After eliminating cases with missing data (5.6% of total sample), a final analytical sample of 24,689 was obtained across the eight surveys.

### Measures of inequality in access to dental services

The approach used to measure lack of access to dental care is based on a dummy variable which takes a value of 1 for children who report having never gone to the dentist and 0 otherwise. Our analysis is undertaken for children aged between 6 and 14, the target population for preventive dental care programmes that was common to all regions (note that some –but not all- regional dental care programmes covered children up to 15 or even 18 but all of the programmes had a common range of 6–14).

### Covariates

As explanatory variables, we have considered demographic, socio-economic and contextual variables for which there is information across the different SNHSs. Particularly, regarding socio-demographic variables, we have considered age and sex. With respect to socio-economic variables, we have just considered occupational social class of the head of the household (i.e. the principal wage earner in the household); regarding education level, its inclusion together with social class, gave problems of multicollinearity, so we finally decided to use social class of the head of the household as proxy for socio-economic status. Regarding contextual variables, for each of the regions, we have considered ratio of dentists per 100,000 population and whether there existed a PADI programme at the corresponding year.

Specifically, we considered the following covariates in our analyses: age group in 3-year intervals (6–8 years old), (9–11 years old) and (12–14 years old); sex; family social class; region of residence; ratio of dentists per 100,000 population at the year of the survey by region; and finally a variable to take into account existing special children’s PADI programme by region. Survey year was also included using one indicator variable each for the eight surveys.

Social class variable is based on the occupation of the principal wage earner in the household. Following Regidor et al. [23] we have assigned each of the occupation categories shown in the different SNHS to one of the following four social classes: upper-level non-manual workers, lower-level non-manual workers, skilled manual workers and unskilled manual workers. For the majority of SNHS (i.e. 1987, 1993, 1995 and 1997), the occupation categories were assigned as follows: upper-level non-manual workers (employers, farmers or managers with six or more employees and professionals); lower-level non-manual workers (self-employed or employers, farmers and managers with five or less employees, supervisors and administrative workers); skilled manual workers; and unskilled manual workers. For the rest of years, there were slight variants. Particularly, for 2003, 2006 and 2011, the categories were similar to those mentioned above but the cut-off number for employees (to distinguish upper and lower non-manual workers) was ten, instead of six. Regarding 2001 a more thorough assignments was undertaken given the much wider information on occupation provided by this particular SNHS.

### Analysis

We estimated pooled logistics regression models to analyse the binary outcomes associated with having never been to the dentist. We estimated our models with regions specified as random effects and as fixed effects in separate models; while the former has the advantage of being more efficient, the latter is often considered to be less biased as all observed and unobserved characteristics of the region that are time-constant are accounted for [24]. Models included interaction terms for time (year of survey) and social class.

The first model, M_1_, is our fixed effect logistic. The second model, M_2_, is the mixed-effect logistic regression containing both fixed effects and random effects at intercept. The results of fixed effects (measures of association) were shown as odds ratios with their 95% confidence intervals. Measures of random effects in mixed-logit included an intra-cluster correlation (ICC). The ICC was calculated by the linear threshold according to the formula used by Snijders and Bosker [25]. Regression diagnostics were used to judge the goodness-of-fit of the model. They included the Akaike’s information criterion (AIC) and Bayesian information criterion (BIC). The statistical significance of covariates was calculated using the Wald test. All significance tests were two-tailed and statistical significance was defined at the 5% alpha level.

## Results

**Figure 1:**
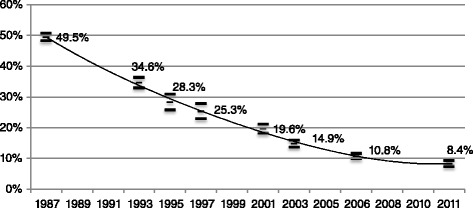
**Proportion of children (6-14 years) who had never visited the dentist 1987-2011.**

Figure 1 shows the proportion of children who had never visited the dentist decreased from 49.5% (CI 48.2%-50.7%) in 1987 to 8.4% (CI 7.4%-9.4%) in 2011, indicating an improvement in children’s access to dental care services as measured in this paper.

Table 1 presents the summary statistics of the variables considered in our analysis for the seven years of the analysed period (1987–2011). Given the dichotomous nature of most of the variables, mean proportions are presented in such a way that the variability in the distribution of the characteristics that explain the probability of not having ever visited the dentist can be appreciated. Apart from the considerable reduction in the proportion of children who had never visited the dentist already noted in Figure 1, Table 1 shows that the demographic characteristics (i.e. gender and age composition), apart from some particular peaks, do not significantly change during the period analysed. Regarding the socioeconomic level of the Spanish population it can be observed, on the one hand, that for the period between 1987 and 2006, there is a relative increase in the weight of the two intermediate social classes to the detriment of the upper non-manual and unskilled manual); this pattern have a change in 2011 when there is a relative increase in the proportion of upper-non manual and unskilled/skilled manual to the detriment of lower non-manual social class, in part, probably due to the effects of the economic crisis.

**Table 1:** **Mean proportions (standard errors) of the covariates used in the analysis**

	**1987**	**1993**	1995-97*	**2001**	**2003**	**2006**	**2011**
Girl	48.2% (0.5)	48.9% (0.5)	48.6% (0.5)	48.0% (0.5)	48.4% (0.5)	49.7% (0.5)	47.2% (0.5)
Age 6–8	32.7% (0.5)	29.9% (0.5)	28.2% (0.5)	31.1% (0.5)	27.0% (0.4)	29.5% (0.4)	31.5% (0.5)
Age 9–11	33.3% (0.5)	34.3% (0.5)	34.4% (0.5)	32.3% (0.5)	32.0% (0.5)	32.4% (0.5)	32.7% (0.5)
Age 12–14	34.0% (0.5)	35.8% (0.5)	37.4% (0.5)	36.6% (.48)	41.0% (.49)	38.2% (0.5)	35.9% (0.5)
Social class							
Upper non-manual	3.6% (0.2)	13.5% (0.4)	16.6% (0.4)	8.6% (0.3)	9.3% (0.3)	10.8% (0.3)	12.0% (0.3)
Lower non-manual	31.5% (0.5)	34.5% (0.5)	31.3% (0.5)	36.7% (0.5)	35.1% (0.5)	36.0% (0.5)	27.8% (0.5)
Skilled manual	38.6% (0.5)	33.5% (0.5)	35.4% (0.5)	45.4% (0.5)	44.7% (0.5)	41.3% (0.5)	46.5% (0.5)
Unskilled manual	26.3% (0.4)	18.5% (0.4)	16.6% (0.4)	9.3% (0.3)	10.9% (0.3)	11.9% (0.3)	13.6% (0.3)
Dental care programme							
PADI	-	3.9% (0.2)	2.6% (0.2)	10.7% (0.1)	12.4% (0.3)	26.4% (0.4)	38.4% (0.5)
Rate of dentists							
per 100,000 inhabitants	17.6	32.34	37.13	44.19	44.58	49.52	60.23
(standard deviations)	(4.1)	(7.7)	(8.9)	(11.1)	(10.7)	(10.6)	(14.9)
N° observations	6046	3162	2453	2861	3718	4845	3079
(missing values)	(230)	(108)	(175)	(364)	(132)	(285)	(181)

Source: Spanish National Health Surveys 1987–2011.

*Note: Due to their relatively small sample sizes, data from the 1995 and 1997 surveys were analysed jointly.

The results of logistic regressions are shown in Table 2. Both fixed effect and mixed effect models present very similar results and statistical significance. Estimated coefficients for the interactions between time and social class and regional dummies in M_1_, and the interactions in M_2_ have been omitted in order to reduce the size of the table and make it clearer.

**Table 2:** **Logistic regression analyses of having never been to the dentist**

	**M**_**1**_ **Fixed effect**	**M**_**2**_ **Fixed-Random effect**
	**Odds ratio (95% Conf. Interval)**	**Odds ratio (95% Conf. Interval)**
Intercept	2.34*** (1.67; 3.29)	1.63*** (1.16; 2.28)
Sex		
Boy	1.00	1.00
Girl	0.91*** (0.86; 0.97)	0.91*** (0.86; 0.97)
Age		
6–8 years old	1.00	1.00
9–11 years old	0.43*** (0.39; 0.46)	0.43*** (0.39; 0.46)
12–14 years old	0.33*** (0.31; 0.36)	0.33*** (0.31; 0.36)
Social class		
Upper non-manual	1.00	1.00
Lower non-manual	1.39* (1.01; 1.90)	1.39** (1.02; 1.90)
Skilled manual	1.52** (1.11; 2.07)	1.51*** (1.11; 2.06)
Unskilled manual	2.35*** (1.71; 3.23)	2.36*** (1.71; 3.24)
Time variables		
Year 1987	1.00	1.00
Year 1993	0.77 (0.52; 1.13)	0.79 (0.63; 1.42)
Year 1995–97	0.51*** (0.33; 0.77)	0.53*** (0.35; 0.80)
Year 2001	0.37*** (0.22; 0.63)	0.40*** (0.24; 0.67)
Year 2003	0.31*** (0.19; 0.52)	0.34*** (0.21; 0.55)
Year 2006	0.20*** (0.12; 0.34)	0.22*** (0.13; 0.36)
Year 2011	0.22*** (0.12; 0.41)	0.25*** (0.14; 0.45)
	Regions have been omitted from table coefficients (see Figure 2)	
	Results for interaction terms for time and occupational class have been omitted from table (see Figure 3)	
Rate of dentists		
per 100,000 inhabitants	0.98*** (0.98; 0.99)	0.98*** (0.97; 0.99)
Dental care programme (PADI)		
Children without PADI	1.00	1.00
Children with PADI	0.82*** (0.71; 0.95)	0.82*** (0.71; 0.94)
		*Random parameters*
		Variance (95% Conf. Interval)
Error variance		
Intercept		0.03 (0.01; 0.06)
*Regression diagnostics*		
Intra-class correlation ICC		1.8%
Log likelihood	−11636.83	−11664.07
AIC	23371.66	23396.14
BIC	23770.43	23672.85
N° of observation	24689	24689

Note: Standard errors in brackets; ***p < 0.01, **p < 0.05, *p < 0.1.

Both M_1_ and M_2_ models present very similar global significance and goodness of fit. According to the AIC criteria, M_1_ would be slightly better than M_2_ whilst according to the BIC criteria, M_2_ would be slightly better than M_1_. However, it can be observed that in the fixed-random effects model, M_2_, the percentage of observed variability in the dependent variable “having never visited the dentist” attributable to being resident in a particular region is very low, ICC = 1.8%. This leads us to consider fixed effects in the variable region of residence and choose the simplest model M_1_, when interpreting the interactions between time and social class variables.

Girls are significantly less likely to have never visited the dentist, showing an odds ratio of 0.91; one explanation that should be further investigated is that girls might be more likely to be flirty than boys and therefore they might go earlier to the dentist. Regarding age, compared with children aged 6–8, those children aged 9–11 and 12–14 are also less likely to have never visited the dentist, with odds ratios of 0.43 and 0.33, respectively. These results seem reasonable as with age it is more likely to have oral health problems and therefore it is more likely to attend the dentist.

The number of dentists per 100,000 inhabitants in the region of residence is significant when explaining the probability of having never visited the dentist (odds ratio of 0.98) indicating that a greater availability of dentists per inhabitants facilitates children’s access to the dentist. In addition, living in a region with a PADI programme significantly reduces the probability of having never visited the dentist (odds ratio of 0.82) compared to those children who live in regions without this sort of programme; in other words having PADIs programmes also improves the probability of access to dental care services. Finally, the trend of the odds ratio of survey year indicators is consistent with the one observed in the descriptive analysis (Figure 1). The probability of never having visited the dentist decreases over time.

**Figure 2:**
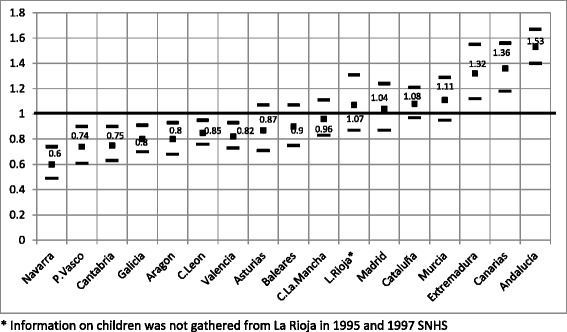
**Logistic regression analysis of the relative odds of having never been to the dentist by regions (M**_**1**_ **Fixed effect model).**

Figure 2 shows the odds ratios (and 95% confidence intervals) by regions. Over the course of this eleven year period, the regions where children have a lower probability of having never visited the dentist are Navarra, País Vasco, Cantabria, Galicia, Aragón, Castilla-León y Valencia. On the other hand, Extremadura, Canarias and Andalucía have a higher probability, indicating a worse access to dentist care services in these regions.

**Figure 3:**
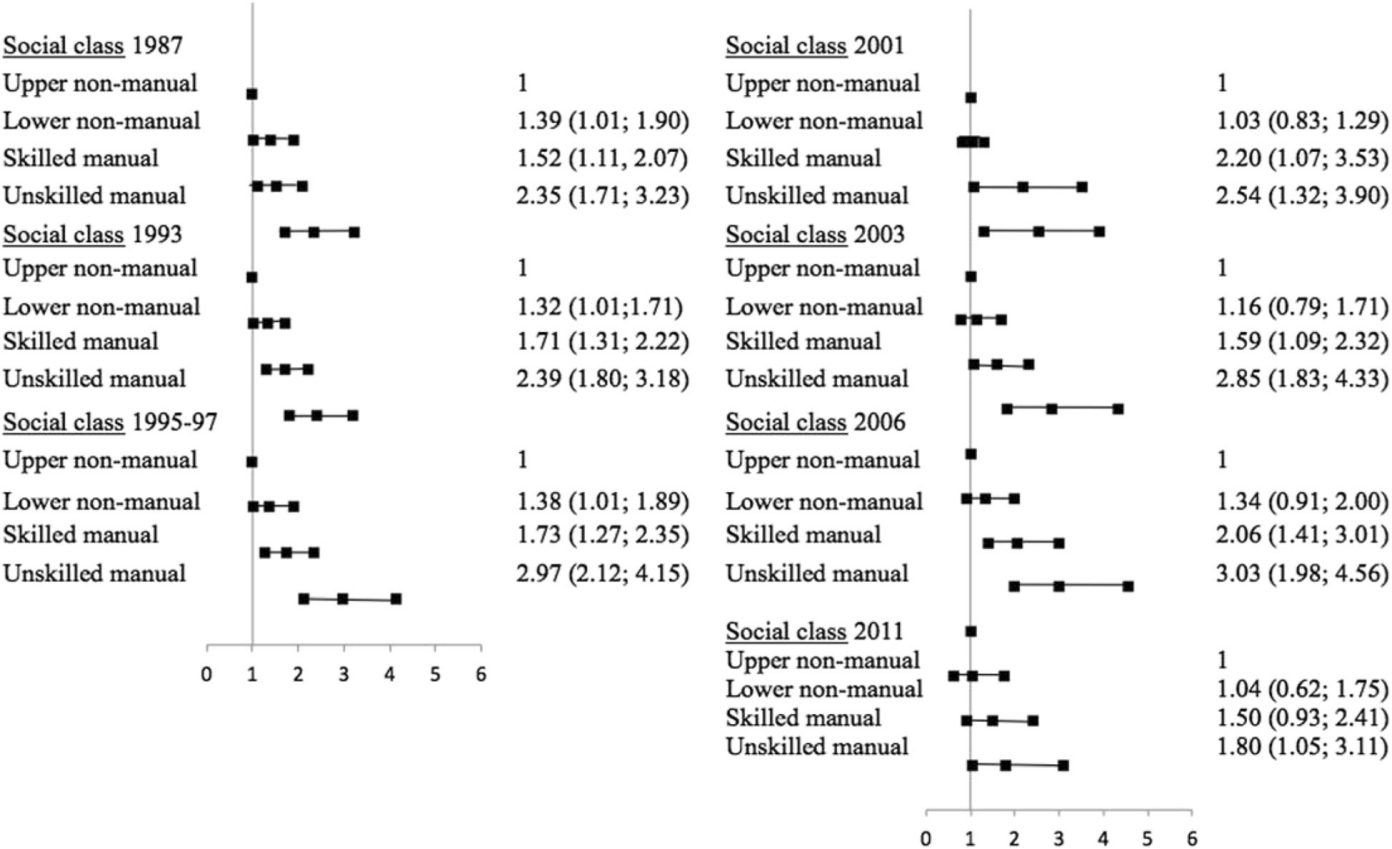
**Logistic regression analysis of the relative odds of having never been to the dentist by social class, by year of survey (M**_**1**_ **Fixed effect model).**

We are also interested in estimating time trends of inequalities in the lack of access to dentists among children between 1987 and 2011. Beta parameters for time dummy variables tell us about the starting place of the time trajectory. However, we have a special interest in estimating the changes in the slope related to the family social class variable during this trajectory. We therefore compared the odds of not visiting the dentist for each social class level compared to visiting the dentist, stratified by year. The estimates are presented in Figure 3 as line graphs showing the proportion of the population experiencing the outcome of interest within each social class level.

In 1987, the probability of not having ever gone to the dentist is more than two times higher for children in the unskilled manual social class than for those in the upper non-manual social class (odds ratio 2.35). And this difference is not reduced significantly throughout the period analysed, rather it increases as in 1993 (odds of 2.39), in 1995–97 (odds of 2.97), in 2001 (odds of 2.54), in 2003 (odds of 2.85) and in 2006 (odds of 3.03), to end in 2011 slightly below than in 1987 (odds ratio of 1.80); the latter might be due to the marginal effect of the last two regional PADI programmes launched in Canarias and Extremadura. So, despite the mean probability of not having ever gone to the dentist falls considerably from 49.5% in 1987 to 8.4% in 2011, the corresponding probability of a child belonging to the lowest social class, instead of approaching that of the highest social class, is maintained or even increased over the period studied.

## Discussion

The results reported in this paper show evidence of a reduction in children’s lack of access to dentists in Spain over the period 1987–2011. However, this reduction has not corrected the socioeconomic inequalities in children’s access to dentists in Spain. In line with other previous studies like Barriuso and Sanz [26] or Tapias-Ledesma et al. [16], we have found a lower access of children belonging to households with low socioeconomic status. In addition, unlike most previous studies (of a cross-section nature), we have done a longitudinal study which has allowed us to show that, far from being reduced, the inequality has remained or even widened over the years.

Regarding our dependent variable “having never visited the dentist”, it clearly indicates a lack of access (either for treatment or just for prevention) to dental care services. To some extent, this represents an advantage with respect to other measures of access (like the probability of visiting or the number of visits to the dentist) that would require a dental care need adjustment. Any child over 5 years old should have visited the dentist for preventive reasons [27]; in other words, every single child is in need of these types of services irrespective of her oral health state. Therefore, not having visited the dentist before -by the time of the survey- corresponds to a lack of access. Not having to adjust for dental care need is a methodological advantage. First, it skips the controversial task of measuring dental care need. Second, it avoids the potential problems of endogeneity of a need variable, present in models of utilisation. Unlike other health care services, a high proportion of dental visits are preventive and if they are effective, then a child’s current oral health heavily depends on past use of services [28]. However, ad-hoc information of need would be relevant to complete our analysis. If information on children’s need for dental care had been available for the period under study, we could have analysed whether evidence elsewhere indicating that need is relatively more concentrated among the lowest socioeconomic groups [6,29] also happens in children’s oral health. Llodra [30] in a study with data from the 2010 Spanish Oral Health Survey shows evidence of a socioeconomic difference in the prevalence of dental caries (approached by the DMFT -Decay Missing Filled Teeth- index) among children aged 12 and 15 (i.e. prevalence increases in lower socioeconomic groups). If this hypothesis is confirmed for children’s oral health need -i.e. inequalities in lack of access that favour higher socioeconomic groups evidenced in our research are accompanied with inequalities in the distribution of need (of the same nature)- then, there would be evidence of the ‘inverse care law’ that has been proven elsewhere for children’s dental care. For instance, Jones [31] analysed the association between the British National Health Service dental registration and deprivation; the system worked as follows: children got free dental treatment under a capitation scheme with an NHS dentist but if children did not attend within 24 months, their registration lapsed and were deleted from the capitation list; he found that registration and lapse rates were significantly associated with social deprivation.

It is also true that having visited the dentist at least once does not guarantee an appropriate access to these services. In order to further discriminate among those who have visited the dentist, additional information on use of dental care services in a given period adjusted by dental care need (ideally distinguishing between treatment and check-ups), would give a more thorough view of access to dental care. However this information was not available for the period analysed.

The main policy change related to children’s dental care in the period analysed has been the implementation of the different regional PADI programmes. However, the extent to which the results obtained in this research are attributable to such programmes or to changes in other demand and supply factors is something that cannot be answered in this paper. In addition, given the heterogeneity in the services provided in different regions, it is likely that there may be different effects among those regions with the infant oral care programmes. For instance, Garcia-Gomez [17] in a study of the País Vasco concludes that such programmes have not had a differential effect on the proportion of those who have never visited the dentist, with respect to regions that did not have it (in a comparative study for the years 1987 and 2001); rather the reduction of this proportion is attributable to a general trend in Spain. Pinilla and González [18] conclude that in comparison with regions without PADIs, the probability of using dental services is greater and independent of household income in those regions with PADI programme. Additionally, Barriuso and Sanz [19] conclude that the use of oral health services is lower than recommended and is positively correlated with the socioeconomic level and with living in regions having a PADI of 10 or more years running.

In our longitudinal study, it has been shown that children living in regions with the PADI programme have greater access to dental care services and this condition has improved over time. An analysis that takes into account not only the time but also the geographical dimension in the application of the PADIs would have been desirable to analyse to what extent the reduction in the proportion of children who have never visited the dentist is due to the effect of the infant oral care programmes or to changes in other factors.

The ultimate aim of children’s dental care is to improve oral health. Elsewhere, it has been shown that the PADI has been effective in improving children’s oral health [32]; however, it would also be desirable to know whether this improvement is also concentrated among those households with higher socioeconomic level, as expected, given the results obtained in our research. In addition, it is interesting to note that the differences in access by regions found in our research are also in line with regional differences in oral health found by Cortes-Martinicorena [13]: according to the children’s dental caries prevalence (approached by the DMFT index), País Vasco and Navarra show lower prevalence whilst Andalucía, Canarias and Extremadura show higher prevalence, just the same distribution that we have had for access in our paper.

If the final aim of a health care policy is to provide health services that can be used by the whole society including all population groups [33], then, the results obtained in this research, particularly those that evidence the persistence of socioeconomic inequalities in the access to dental care services, should be taken into account by health authorities when designing (or improving current) children’s dental health programmes. It might be of help to analyse first the reasons why those children belonging to lower socioeconomic levels experience a greater lack of access to dental care services, that is, whether this inequality it is driven by demand factors (i.e. those related to the socioeconomic level or socio-demographic characteristics) by supply factors (i.e. distance to the point of consumption, difficulties in getting appointments, waiting times, information in those more deprived areas, etc.) or by a combination of both Nevertheless, any reference to the policy implications from the equity point of view must take into account that the aim of our study was to analyse socioeconomic inequalities in the lack of access to any type of children’s dental care (both publicly and privately financed) rather than a study of equity in access to children’s dental care services.
